# Sarcolemmal Excitability, M-Wave Changes, and Conduction Velocity During a Sustained Low-Force Contraction

**DOI:** 10.3389/fphys.2021.732624

**Published:** 2021-10-15

**Authors:** Javier Rodriguez-Falces, Nicolas Place

**Affiliations:** ^1^Department of Electrical and Electronical Engineering, Public University of Navarre, Pamplona, Spain; ^2^Institute of Sport Sciences, University of Lausanne, Lausanne, Switzerland

**Keywords:** submaximal contraction, low-level contraction, sustained contraction, membrane excitability, conduction velocity, peripheral fatigue, neuromuscular propagation

## Abstract

This study was undertaken to investigate whether sarcolemmal excitability is impaired during a sustained low-force contraction [10% maximal voluntary contraction (MVC)] by assessing muscle conduction velocity and also by analyzing separately the first and second phases of the muscle compound action potential (M wave). Twenty-one participants sustained an isometric knee extension of 10% MVC for 3min. M waves were evoked by supramaximal single shocks to the femoral nerve given at 10-s intervals. The amplitude, duration, and area of the first and second M-wave phases were computed. Muscle fiber conduction velocity, voluntary surface electromyographic (EMG), perceived effort, MVC force, peak twitch force, and temperature were also recorded. The main findings were: (1) During the sustained contraction, conduction velocity remained unchanged. (2) The amplitude of the M-wave first phase decreased for the first ~30s (−7%, *p*<0.05) and stabilized thereafter, whereas the second phase amplitude increased for the initial ~30s (+7%, *p*<0.05), before stabilizing. (3) Both duration and area decreased steeply during the first ~30s, and then more gradually for the rest of the contraction. (4) During the sustained contraction, perceived effort increased fivefold, whereas knee extension EMG increased by ~10%. (5) Maximal voluntary force and peak twitch force decreased (respectively, −9% and −10%, *p*<0.05) after the low-force contraction. Collectively, the present results indicate that sarcolemmal excitability is well preserved during a sustained 10% MVC task. A depression of the M-wave first phase during a low-force contraction can occur even in the absence of changes in membrane excitability. The development of fatigue during a low-force contraction can occur without alteration of membrane excitability.

## Introduction

The excitability of the muscle fiber membrane (i.e., the ability to generate and propagate transmembrane action potentials) requires the maintenance of steep chemical gradients for Na^+^ and K^+^ across the sarcolemma (Nielsen and Clausen, 2000). Sarcolemmal membrane excitability has been extensively studied during maximal voluntary contractions (MVCs), but less so during low-force contractions. It has been suggested that contractions below 15% MVC might be sustained “indefinitely” (beyond 45min; Bigland-Ritchie and Woods, 1984; Burnley et al., 2012). This prolonged endurance time may be due to the fact that, at such low contraction levels, blood flow is sufficiently high to maintain K^+^ homeostasis, as previously suggested (Sjøgaard et al., 1988). More specifically, as a low-force contraction is sustained, the impulse-mediated efflux of K^+^ would act to increase the concentration of this ion in the extracellular medium, but such increase would be counteracted by the diffusion of K^+^ into the capillaries and removal by the bloodstream (Jensen et al., 2004; Clausen, 2011). However, despite the evidence suggesting that K^+^ homeostasis is maintained during a sustained low-force contraction, it is not clear whether membrane excitability is preserved during such contractions.

Adjustments in sarcolemmal excitability during a contraction are commonly studied by observing the changes of the compound muscle action potential (M wave). Interestingly, the studies assessing changes in M-wave size induced by low-force contractions have yielded consistent results: In particular, M-wave amplitude and area were found to remain unchanged after a 43-min contraction at 15% MVC in the *biceps brachii* (Søgaard et al., 2006) and also after a 70-min contraction at 5% MVC in the same muscle (Smith et al., 2007). However, the above-mentioned studies suffered from three major methodological limitations in their attempt to investigate sarcolemmal excitability. First, in none of the previous studies were the measures of M-wave amplitude accompanied by data on muscle fiber conduction velocity. And the velocity of impulse conduction is probably the parameter that most faithfully reflects the adjustments in membrane excitability (Cupido et al., 1996; Fortune and Lowery, 2009). Second, these studies only assessed the gross parameters of the M wave: i.e., its peak-to-peak amplitude and/or total area. However, the sole analysis of the whole M wave has been shown to be inadequate and misleading to identify and interpret changes in sarcolemmal excitability: a separate analysis of the first and second M-wave phases is mandatory (Rodriguez-Falces and Place, 2017a,b, 2018). In particular, because the M-wave second reflects the extinction of action potentials at the tendon, this phase is highly sensitive to positional changes of the recording electrodes relative to the muscle-tendon complex (Rodriguez-Falces and Place, 2014). Thus, it would be interesting to test to what extent the decrease in fascicle length at the initial part of a submaximal contraction (Mademli and Arampatzis, 2005) influences the magnitude of the M-wave second phase. Third, most of previous works only reported the changes in M-wave amplitude from before to after the prolonged contraction, thereby missing the changes occurring during exercise. However, the time course of changes during the contraction may be crucial to interpret the possible adjustments in membrane excitability.

In addition to the above methodological flaws, the M wave has its own limitations as an index of membrane excitability. The reason is that M-wave amplitude may be influenced by several factors other than just the transmembrane action potential (for a review, see Rodriguez-Falces and Place, 2018). Specifically, during a sustained submaximal contraction, the decrease in muscle fascicle length (Mademli and Arampatzis, 2005) would cause the muscle to operate at a shorter length (Maganaris et al., 2002), a factor which could increase the amplitude of the M-wave second phase (Rodriguez-Falces and Place, 2014). Another factor, the increase in intramuscular temperature during a contraction, is known to have a depressing effect on M-wave amplitude (Rutkove, 2000). Another aspect to consider is that the recruitment threshold of axons may increase during a submaximal contraction (Vagg et al., 1998), which could decrease M-wave size.

It has been shown that sustained low-force contractions induce muscle fatigue, as evidenced by a decrease in MVC force immediately after exercise (Kouzaki et al., 2004; Søgaard et al., 2006; Smith et al., 2007). This fatigue is not only due to central mechanisms, but also to peripheral factors as shown by a decrease in the peak twitch force (Blangsted et al., 2005; Søgaard et al., 2006; Smith et al., 2007). In these studies, the amplitude of the M waves, as measured from peak to peak, remained unchanged, which prompted the authors to conclude that peripheral fatigue was not due to an impairment in membrane excitability. However, it is important to measure conduction velocity and assess individually the first and second M-wave phases to evaluate sarcolemmal excitability.

This study investigated three research questions: (1) Is sarcolemmal excitability preserved during prolonged low-force contractions? (2) How do the amplitudes of the first and second M-wave phases vary during such contractions? (3) Does sarcolemmal excitability have any role in the development of fatigue in such contractions? These questions were addressed by examining separately the first and second phases of the M wave during the course of a 3-min contraction sustained at 10% MVC in the quadriceps, and also by assessing the changes in muscle fiber conduction velocity. The possibility that the low-force contraction induced muscle fatigue was investigated by assessing the maximal voluntary and evoked force before and after exercise. Based on the results of Sjøgaard et al. (1988) on K^+^ homeostasis, we hypothesized that sarcolemmal excitability would be preserved, and thus conduction velocity would not decrease throughout the low-force contraction. Based on the studies of Mademli and Arampatzis (2005), which showed a significant decrease in fascicle length at the initial part of a sustained submaximal contraction (at 80s during a 400-s contraction), we expected that M-wave parameters would change early in the 3-min contraction.

## Materials and Methods

### Participants

Twenty-one male participants aged between 22 and 31years (mean±SD: 25±3years) with no known history of neurological or musculoskeletal impairments participated in this study. Their average height and body mass were 177±6cm and 70±6kg, respectively. The experiments were conducted in accordance with the Declaration of Helsinki and were approved by the research ethics board of the Public University of Navarra, Spain (PI-010/21). All participants gave written informed consent to participate in the study after receiving a detailed explanation of the purposes and risks of the study.

### Experimental Setup

**Figure 1:**
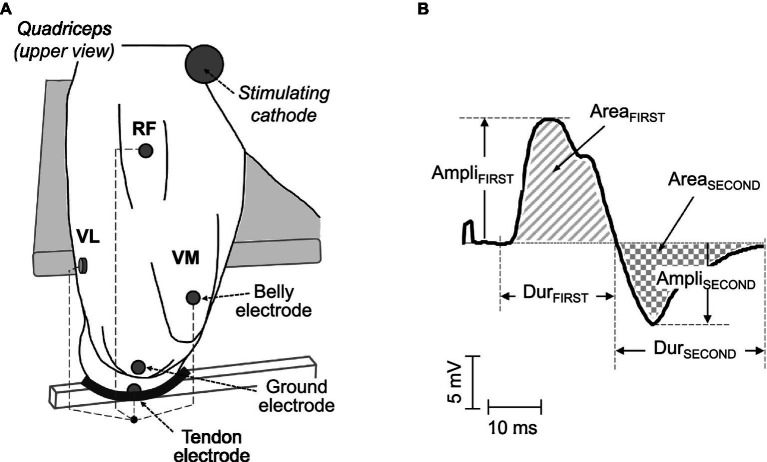
**(A)** Electrode arrangements for the recording of surface electromyographic (EMG) signals in the *quadriceps* muscles. In each muscle, a pair of electrodes was placed in a belly-tendon configuration: the “belly” electrode was located over the innervation zone, whereas the “tendon” electrode was positioned over the patellar tendon. **(B)** Example of a typical belly-tendon M wave, along with its parameters: amplitude, duration, and area of the first (Ampli_FIRST_, Dur_FIRST_, and Area_FIRST_) and second (Ampli_SECOND_, Dur_SECOND_, and Area_SECOND_) phases.

Experiments were carried out on the *quadriceps* muscle and consisted on applying electrical stimulation while the participants performed an isometric knee extension contraction at 10% MVC force (75±9N, Figure 1). Participants were seated comfortably on a custom-built chair with a knee angle of 90° and a trunk-thigh angle of 100°. Extraneous movements of the upper body were prevented by two crossover shoulder harnesses and a belt across the lower abdomen. Quadriceps force was recorded during the voluntary isometric contractions using a strain gauge (STS, SWJ, China, linear range: 0–2,452N, sensitivity 2mV/V and 0.0017V/N) that was attached to the chair and securely strapped to the ankle with a custom made mold. The force signal (knee extension) was sampled at 1,000Hz using an analog-to-digital conversion system (MP150; BIOPAC, Goleta, CA, United States).

### Localization of the Innervation Zone and the Muscle Fibers’ Direction

The innervation zone and muscle fibers’ direction were identified in each muscle using a dry linear array of 16 electrodes (5mm inter-electrode distance) during gentle isometric contractions. The array was connected to a multichannel amplifier (OT Bioelettronica, Torino; bandwidth 10–500Hz) and electromyographic (EMG) signals were recorded in single-differential (bipolar) configuration. The location of the innervation zone was determined by observing the channel of the array showing minimum amplitude or phase reversal (Masuda et al., 1985). The direction of the muscle fibers was identified by choosing the orientation of the array that yielded optimal propagation of action potentials between the innervation zone and tendon regions (Farina et al., 2002).

### Electromyographic Recordings

Surface EMG potentials were recorded from the *vastus lateralis* (VL), *vastus medialis* (VM), and *rectus femoris* (RF) muscles. Since these three muscles are innervated by the femoral nerve, they were activated “at once” by delivering electrical stimuli to this nerve. EMG potentials were recorded using circular Ag/AgCl surface electrodes (Kendall Meditrace 100), with a recording diameter 10mm. Before electrode placement, the skin was adequately prepared (shaving, light abrasion with sandpaper, and cleaning with rubbing alcohol) to reduce the impedance at the skin-electrode interface. Surface EMG signals were amplified (bandwidth: 10–1,000Hz) and digitized (sampling frequency of 5kHz) using an analog-to-digital conversion system (MP150; BIOPAC, Goleta, CA).

In each muscle, the recording electrodes were placed in a “belly-tendon” montage, as illustrated in Figure 1. Specifically, the “belly” electrode was located over the innervation zone of each muscle (see above), whereas the “tendon” electrode was placed over the patellar tendon. The ground electrode was positioned over the ipsilateral patella, as depicted in Figure 1. The M waves recorded under the belly-tendon montage can be considered as monopolar M waves since the tendon electrode was placed on an electrically non-active site of the body and thus recorded a negligible potential.

To measure conduction velocity, a linear adhesive electrode array of eight electrodes (5mm inter-electrode distance, OT Bioelettronica, Torino, Italy) was placed adjacent to the belly electrode in the VL and VM muscles. The array was placed distally with respect to the innervation zone of each muscle (where unidirectional propagation of action potentials occurred) and oriented parallel to the muscle fibers’ direction. To ensure proper electrode-skin contact, electrode cavities of the array were filled with 20–30μl of conductive paste. The surface EMG signals were amplified, sampled at 2,048Hz, band-pass filtered (3dB bandwidth, 10–500Hz), and converted to digital data by a 12-bit A/D converter (EMG-USB, OT Bioelettronica, Torino, Italy).

### Stimulation Procedure

The femoral nerve was stimulated using single rectangular pulses (1-ms duration) delivered by a high-voltage constant current stimulator (DS7AH; Digitimer, Hertfordshire, United Kingdom). The cathode was a circular (5cm diameter) self-adhesive electrode (Dermatrode, American Imex, Irvine, CA) positioned in the femoral triangle, 3–5cm below the inguinal ligament. The anode was a large (5×10cm) rectangular self-adhesive electrode (Compex, Ecublens, Switzerland) located over the gluteal fold. To determine the stimulus intensity corresponding to full motor unit recruitment, stimulus strength was gradually increased until a plateau in the M-wave amplitude of the VL, VM, and RF muscles was observed. This level of intensity was then further increased by 20% to ensure that the stimulation remained supramaximal throughout the experimental session.

### Experimental Protocol

**Figure 2:**
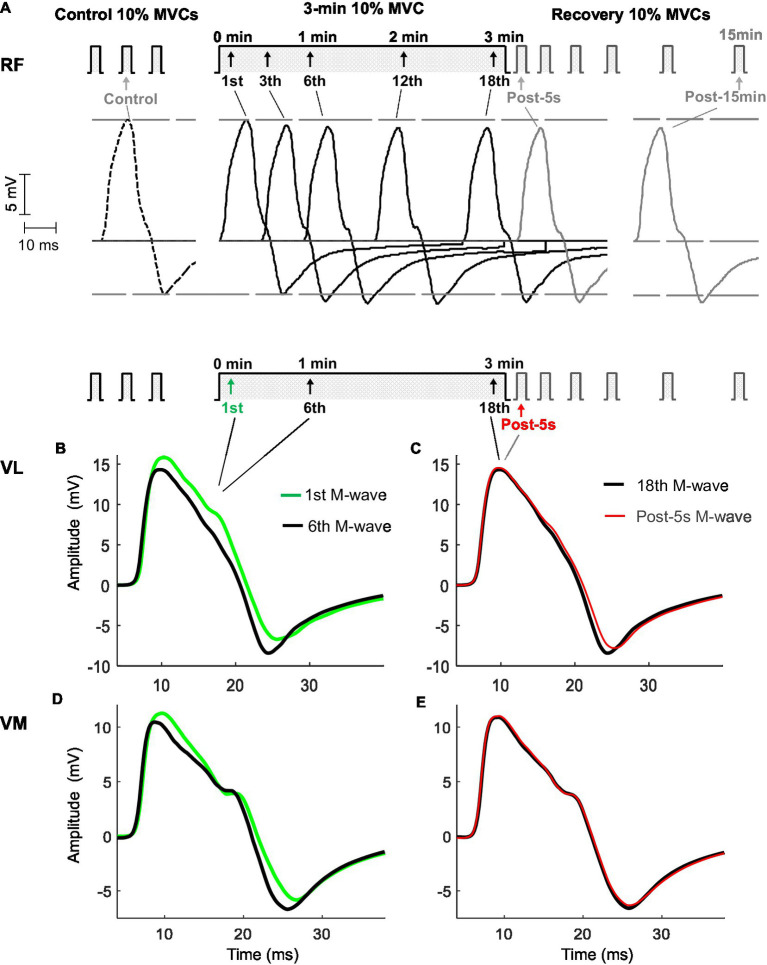
Representative examples of M waves recorded from the *rectus femoris* (top panel, **A**), *vastus lateralis*, and *vastus medialis* (bottom panel, **B,C,D,E**) before (control), during, and after (post) the 3-min sustained contraction at 10% maximal voluntary contraction (MVC) in one participant. In the top panel **(A)**, M waves are shown in chronological sequence to better appreciate the changes in the amplitude of the first and second phases throughout the protocol. Note that Ampli_FIRST_ decreased during the first minute and stabilized thereafter, while Ampli_SECOND_ increased for 1min, before stabilizing. In the bottom panel **(B,C,D,E)**, M waves are shown in a superimposed fashion to better recognize the changes in M-wave duration the protocol. M-wave duration decreased during the 3-min contraction.

The protocol consisted of three sequential stages: (1) initial control measurements, (2) a prolonged contraction (3min at 10% MVC), and (3) a recovery period (see below for details and also Figure 2, top panel). Participants were well familiarized with the experimental procedures as they all have been involved in previous experiments. Participants were instructed not to exercise with their legs or to engage in heavy physical work for a day beforehand.

The experiment started with participants performing three brief (4s) control MVCs, with 3min of rest in between. The peak forces from these three MVCs were averaged to determine each subject’s MVC force: this average maximal force was used to calculate the “target” force corresponding to 10% MVC. After 5min of rest, three brief (4s) contractions at 10% of MVC were performed to provide control measures. To do this, a single supramaximal stimulus was delivered at the mid-point of each 10% MVC bout to evoke the M wave superimposed on the ongoing voluntary effort. The average of these three superimposed M waves was calculated to extract control M-wave values (see below). Finally, three single supramaximal stimuli were applied with the muscle fully relaxed to obtain the control twitches.

The prolonged contraction consisted of 3min of isometric knee extension at 10% MVC. The choice of this contraction duration (3min) was because the main changes in M-wave parameters were expected to occur early in the contraction due to alterations in muscle architectural properties (Mademli and Arampatzis, 2005), while pilot experiments showed that this duration was long enough to induce significant muscle fatigue. All participants were able to maintain the contraction for this period. During the contraction, participants were instructed to match the target force level of 10% MVC, digitally displayed on a computer monitor (participant’s force was also displayed on the monitor). Single shocks were applied every 10s during the contraction, and the resulting superimposed M waves were recorded (Figure 2).

During recovery, participants were divided into two groups: This allowed us to assess both electrical (M-wave parameters) and mechanical (MVC and twitch force) variables. In the first group (10 participants), after cessation of the 3-min contraction, six brief (4s) contractions at 10% MVC were performed at 5s, 1min, 2min, 5min, 10min, and 15min, while a single shock was superimposed during each brief contraction. In the second group (11 participants), after cessation of the 3-min contraction, participants performed three brief (4s) MVCs, at 7s, 3min, and 15min into the recovery. In addition, for this second group, a single supramaximal stimulus was delivered at rest 5s after the cessation of the 3-min contraction.

### Temperature Recordings

Skin surface temperature was measured during the course of the low-force contraction using a fast-response thermistor (SS6L, BIOPAC, response time 0.6s, accuracy ±0.1°C). The temperature probe was located close to the surface electrodes over the *rectus femoris*. During the experiments, room temperature was kept at 22±0.2°C.

### Rating of Subjective Effort

During the prolonged contraction, participants were asked every 30s to score the perceived effort required to produce the target (10% MVC) force on a modified Borg scale from 0 (“nothing”) to 10 (“maximal”; Borg et al., 1985).

### Data Analysis

For each M-wave potential, the amplitude, duration, and area of the first (Ampli_FIRST_, Dur_FIRST_, and Area_FIRST_) and second (Ampli_SECOND_, Dur_SECOND_, and Area_SECOND_) phases were computed as in previous studies (see Figure 1 and Rodriguez-Falces and Place, 2017a,b). The onset for Dur_FIRST_ was determined by a deviation greater than 2 SDs of the baseline. The onset for Dur_FIRST_ was determined by a deviation greater than 2 SDs of the baseline noise from the baseline, whereas the end-point corresponded to the baseline-crossing point. This crossing point marked the onset of the second phase. The end-point for Dur_SECOND_ was determined by a deviation less than 2 SDs of the baseline noise from the baseline. The area parameters were calculated as the integral of the absolute value of the M wave over the above-defined phases. Ampli_PP_ was computed as the sum of Ampli_FIRST_ and Ampli_SECOND_. Area_TOTAL_ was calculated by adding the areas of the first and second phases. Dur_PP_ was computed as the time interval between the first and second peaks of the M wave. For the quadriceps twitch, the peak force was measured.

The above M-wave and twitch parameters were calculated using custom-written scripts implemented in MATLAB (MathWorks, Natick, MA). The control “reference” values for the above M-wave and twitch parameters were extracted from the M waves and twitches evoked before the prolonged contraction. All the M-wave parameters recorded during the 3-min contraction, and the subsequent brief contractions were expressed as percentage of the control responses.

Estimation of conduction velocity was performed using the multidip approach (Farina and Negro, 2007) with channels 2–5 of the electrode array (as this approach uses only four consecutive EMG channels). The multidip method is based on the use of a regression analysis of the spatial and temporal frequencies of multiple dips introduced into the EMG power spectrum through the application of a set of spatial filters (Farina and Negro, 2007). The estimation of the EMG activity for each muscle was determined as the root mean square (RMS) value over a window of 0.5s duration (Rozand et al., 2015). The average EMG RMS from the VL, VM, and RF was calculated.

### Statistics

Kolmogorov–Smirnov tests confirmed that each parameter analyzed in the current study was normally distributed. The changes in the M-wave parameters, conduction velocity, temperature, and participants’ perceived effort during the 3-min contraction were investigated with a one-way repeated-measures ANOVA (Time). A two-way repeated-measures ANOVA (time×muscle) was adopted to investigate the changes in EMG RMS. The changes in the M-wave parameters during the 15-min recovery were investigated with a one-way repeated-measures ANOVA (Time). Possible changes in the MVC force and peak twitch force from before to after the 3-min contraction were examined using a one-way ANOVA. When main effects were significant, Student–Newman–Keuls *post hoc* tests were conducted. Significance was set at *p*<0.05. Data were presented as mean±SD in the text and tables and as mean±SE in the figures.

## Results

### Changes in MVC Force and Twitch Peak Force

After cessation of the 3-min contraction, 11 of 21 participants performed three brief MVCs at 7s, 3min, and 15min into the recovery. The MVC force measured 7s after the contraction declined by 9±3% (*p*<0.05, range 4.1–12.7) of its value during the control MVCs, and MVC force returned to control values after 3min (*p*>0.05). With regard to the “resting” twitch evoked 5s after the cessation of the 3-min contraction, it had a peak force significantly decreased compared to the control twitch (92±6% vs. 102±7%, *p*<0.05).

### Conduction Velocity, Muscle Temperature, EMG, and Perceived Effort During the Low-Force Contraction

**Figure 3:**
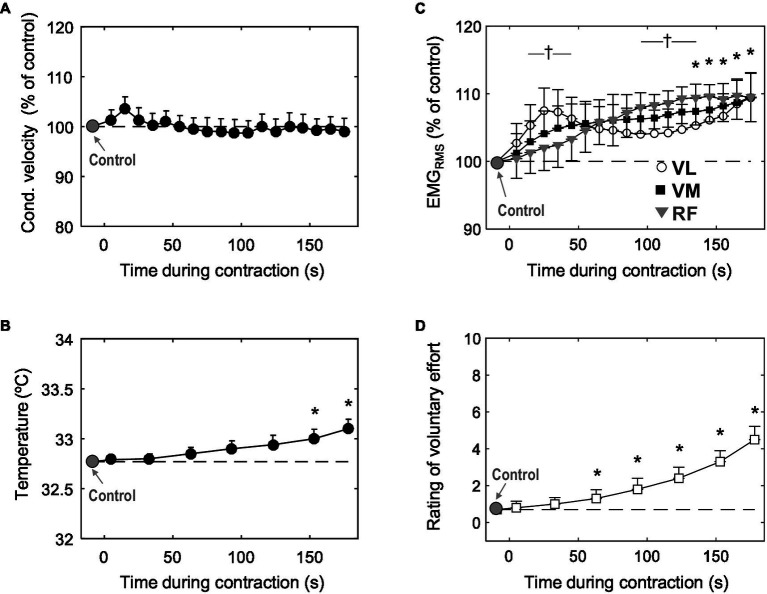
Time course of changes in muscle fiber conduction velocity **(A)** muscle skin temperature **(B)** mean EMG_RMS_ from the vastus lateralis, vastus medialis, and rectus femoris **(C)**, and participants’ perceived effort **(D)** during the 3-min sustained contraction at 10% MVC. Data are reported as mean±SE (*n*=21) and, in plots **(A)** and **(B)**, data are expressed as percentage of control values. ^*^Significant difference with control (*p*<0.05). ^†^Significant difference in EMG_RMS_ between VL and RF (*p*<0.05).

The left panel of Figure 3 shows the average changes in conduction velocity (a) and muscle skin temperature (b) during the low-force contraction. It can be seen that conduction velocity remained similar to control values for the entire duration of the contraction (*p*>0.05). Average absolute values of conduction velocity were 4.1±0.8m/s. Muscle skin temperature increased very slowly and gradually during the contraction, and this increase became significant only during the last minute (*p*<0.05).

The right panel of Figure 3 shows the average changes in EMG_RMS_ (c) and participants’ perceived effort (d) during the contraction. Voluntary EMG_RMS_ increased significantly (*p*<0.05) for the three muscles tested. In the VM and RF, the increase in EMG_RMS_ occurred progressively throughout the contraction, whereas for the VL, EMG_RMS_ increased rapidly for the first ~30s, when it reached a peak (*p*<0.05), after which it decreased slightly for the about minute and then increased again for the remainder of the contraction (significant time×muscle interaction, *p*<0.05). Perceived effort increased significantly during the task (*p*<0.05): more specifically, at the end of the contraction, subjective effort was increased fivefold with respect to the value at the beginning.

### Representative M Waves Before, During, and After the Low-Force Contraction

Figure 2 shows typical examples of M waves recorded from the RF (top panel) and VL and VM (bottom panels) muscles evoked at various times before, during, and after the sustained low-force contraction. In the top panel, it can be seen that Ampli_FIRST_ and Ampli_SECOND_ changed in opposite directions throughout the contraction. Specifically, Ampli_FIRST_ diminished during the first minute (from the first to the sixth response) and then stabilized thereafter (from the sixth to the 18th response), whereas conversely, the Ampli_SECOND_ enlarged during the first minute, before reaching a plateau. The opposing changes in the first and second phases were also observed in the VL (Figure 2B) and VM (Figure 2E) muscles. Noteworthy, Ampli_FIRST_ and Ampli_SECOND_ did not change from the M wave evoked at the end of the 3-min contraction (18th) to the M wave elicited after 5s of rest (Post-5s; Figures 2A,C,E).

To better appreciate the changes in the duration of the M wave, various responses evoked during and after the 3-min contraction are plotted in superimposed fashion (bottom panel). It can be seen that M-wave duration decreased noticeably during the first minute of the contraction (from the first to the sixth response). Noteworthy, M-wave duration increased only slightly from the M wave evoked at the end of the 3-min contraction (18th response) to the M wave elicited after 5s of rest (Post-5s).

### M-Wave Parameters During the Low-Force Contraction

**Figure 4:**
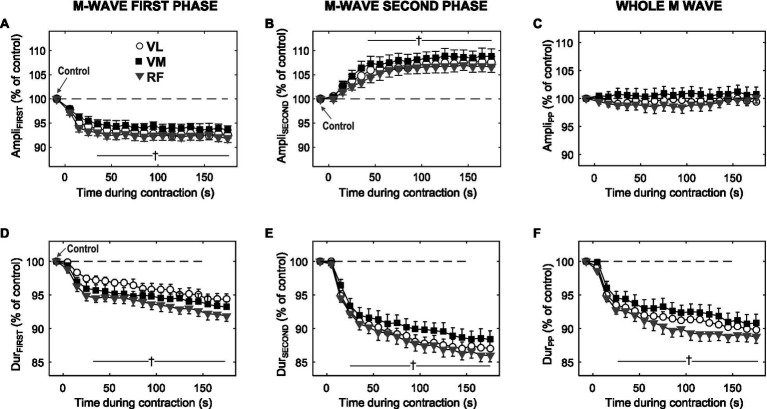
Time course of changes in amplitude and duration for the first phase (Ampli_FIRST_ and Dur_FIRST_, respectively), and the second phase (Ampli_SECOND_ and Dur_SECOND_, respectively) of the M wave, and also for the whole M wave (Ampli_PP_ and Dur_PP_, respectively), during the 3-min sustained contraction at 10% MVC. All data are expressed as percentage of control values and reported as mean±SE (*n*=21). ^†^Significant difference with control for all muscles (*p*<0.05).

Figure 4 shows the average changes in the amplitude and duration of the first phase (first column), and second phase (second column) of the M wave, and also for the whole M wave (third column), during the sustained 10% MVC forcetask. It can be seen that, for all muscles tested, Ampli_FIRST_ decreased significantly during the first ~30s of the contraction (*p*<0.05, Table 1) and then levelled off for the rest of the contraction (Figure 4A). In contrast, Ampli_SECOND_ increased rapidly during the first ~30s of the contraction (*p*<0.05, Table 1) and then stabilized at a plateau for the rest of the task (Figure 4B). As a result, Ampli_PP_ remained unchanged throughout the 3-min contraction (Figure 4C).

**Table 1:** Control and peak values of the amplitude and area of the first and second phases of the M wave during the 3-min sustained contraction at 10% MVC, together with the times at which they occurred.

	Ampli_FIRST_				Ampli_SECOND_			
Muscle	Control (mV)	Minimum (mV)	Change (%)	Time (s)	Control (mV)	Maximum (mV)	Change (%)	Time (s)
*Vastus lateralis*	12.8±3.3	11.9±2.2	−6.8±1.3	175±11	7.1±2.2	7.5±2.2	+6.8±1.8	135±15
*Vastus medialis*	11.9±3.5	11.1±2.2	−6.1±1.2	145±16	9.3±2.4	10.0±2.0	+7.6±2.0	145±14
*Rectus femoris*	13.4±3.9	12.4±2.4	−7.8±1.5	175±12	5.8±1.4	6.1±2.3	+6.2±1.6	165±12
	**AreaFIRST**				**AreaSECOND**			
**Muscle**	**Control (mV∙ms)**	**Minimum (mV∙ms)**	**Change (%)**	**Time (s)**	**Control (mV∙ms)**	**Minimum (mV∙ms)**	**Change (%)**	**Time (s)**
*Vastus lateralis*	123.6±38	110.0±10	−11.0±2.2	175±10	106.2±6.7	91.5±8.5	−13.8±12	175±9
*Vastus medialis*	118.5±35	104.4±10	−11.9±2.3	175±12	102.5±6.9	91.9±11.4	−10.3±15	175±12
*Rectus femoris*	159.3±9.5	136.9±12	−14.2±2.6	175±11	125.6±5.3	105.0±10.8	−16.4±17	175±13

All values are expressed as mean±SD. All values were significantly different from controls (*p*<0.05, *n*=21).

Unlike the amplitude parameters, Dur_FIRST_ (plot d) and Dur_SECOND_ (plot e) behaved in a similar manner during the contraction: both parameters decreased rapidly and significantly during the initial ~30s (*p*<0.05), and subsequently continued decreasing more gradually for the remainder of the contraction. The time course of changes in Dur_PP_ followed the same trend as that of Dur_FIRST_ and Dur_SECOND_. Although not shown in Figure 4, the changes in Area_FIRST_ and Area_SECOND_ were in all aspects similar to those in Dur_FIRST_ and Dur_SECOND_, respectively.

### Changes in the M-Wave Parameters During Recovery

**Figure 5:**
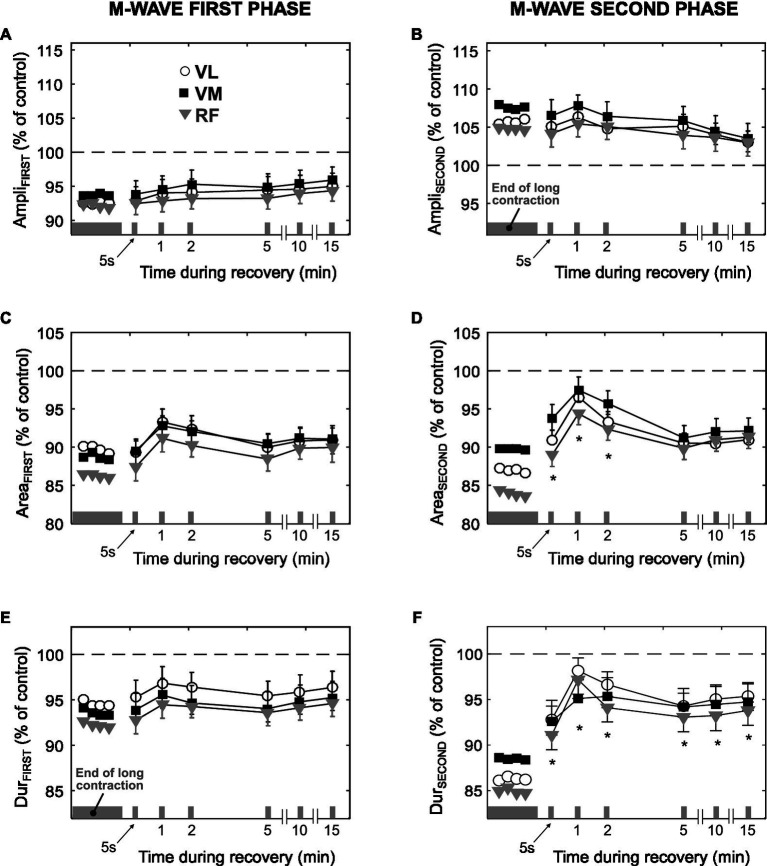
Time course of changes in the amplitude, area, and duration of the first phase (Ampli_FIRST_, Area_FIRST_, and Dur_FIRST_, respectively) and second phase (Ampli_SECOND_, Area_SECOND_, and Dur_SECOND_, respectively) of the M wave during the recovery after the long low-force contraction. All data are expressed as percentage of control values (dashed line) and reported as mean±SE (*n*=10). ^*^Significant difference compared to the last M wave evoked during the 3-min contraction (*p*<0.05).

After cessation of the 3-min contraction, 10 of 21 participants performed six brief 10% MVC bouts at various time points, while a single shock was superimposed during each brief contraction. Figure 5 shows the average values of M-wave parameters during such brief contractions. The values of Ampli_FIRST_, Area_FIRST_, and Dur_FIRST_ after 5s of rest were similar to those corresponding to the last M wave (18th) elicited during the 3-min contraction (*p*>0.05, Figures 5A,C,E). The value of Ampli_SECOND_ remained unchanged after 5s of rest (*p*>0.05, Figure 5B), whereas the values of Area_SECOND_ and Dur_SECOND_ increased after 5s of rest (*p*<0.05, Figures 5D,F). Neither of the M-wave parameters returned to control values after 15min of rest (*p*<0.05).

## Discussion

The present study investigated possible changes in sarcolemmal excitability during a 3-min contraction of the knee extensors sustained at 10% MVC. The main findings were: (1) Both maximal voluntary force and resting peak twitch force decreased after the low-force contraction; (2) Conduction velocity remained unchanged during the sustained contraction; (3) The amplitude of the M-wave first phase decreased for the first ~30s, and stabilized thereafter, whereas the second phase amplitude increased for the initial ~30s, before stabilizing; and (4) Both duration and area decreased steeply during the first ~30s, and then more gradually for the rest of the contraction.

### Muscle Fatigue Induced by the Prolonged Contraction: Central and Peripheral Mechanisms

The decline in maximal voluntary force (−9%) observed immediately after the 3-min 10% MVC task indicate that such contraction produced muscle fatigue, in agreement with previous observations during low-force contractions (Søgaard et al., 2006; Smith et al., 2007). Part of this loss of force was due to peripheral factors, as there was a decrease in the amplitude of the resting twitch (~10%) from before to after the contraction. This peripheral fatigue could not be due to an impairment of fiber membrane processes, as both the present results and those of Sjøgaard et al. (1988) indicate that sarcolemmal excitability was preserved throughout the contraction. Therefore, the depression of twitch force was probably caused by altered Ca^2+^ handling and/or impairment at the cross bridges level (Rassier and Macintosh, 2000).

The deficit observed in MVC force probably has a central component. This conclusion is based on the mismatch between the changes in voluntary EMG_RMS_ and those in participants’ perceived effort: indeed, we observed that EMG required to maintain the target force increased only slightly (~10%) from the beginning to the end of the contraction, whereas the participants’ effort increased fivefold. This disproportionate increase in perceived effort compared to EMG has been also observed during an isometric elbow flexion contraction at 5% MVC (Smith et al., 2007) and could be due to several mechanisms: (1) the active motoneurons became harder to drive when they are subjected to long, repetitive activation (Kernell and Monster, 1982; Johnson et al., 2004; Finn et al., 2018) and (2) the decline in muscle spindle discharge rates during fatiguing contractions would reduce facilitation to motoneurons (Macefield et al., 1991).

### Conduction Velocity

We observed that muscle fiber conduction velocity remained stable throughout the 10% MVC task in the *vastus lateralis*, an observation that apparently contradicts the findings of Arendt-Nielsen et al. (1989), who found that “conduction velocity rises as the contraction progresses” for a contraction of the same intensity in the same muscle. This discrepancy may be explained by the fact that, in the case of Arendt-Nielsen et al. (1989), the contraction was maintained for 400s, i.e., almost twice as long as our case, 180s. Therefore, it is tentatively suggested that, in the experiments of Arendt-Nielsen, the increase in conduction velocity started to occur after the third minute of the contraction.

The absence of changes in conduction velocity may appear surprising in view of the fact that voluntary EMG_RMS_ increased during the contraction. Indeed, the increase in EMG_RMS_ suggests that the number of active motor units increased to maintain the target force (Fallentin et al., 1993), since at low force levels, the discharge rate of the active motor units tends to decrease (Kamo and Morimoto, 2001; Riley et al., 2008). Thus, conduction velocity may have remained stable due to two factors: (1) very few additional motor units were recruited (in fact, EMG_RMS_ only increased by 10%), and (2) the newly recruited units had a conduction velocity roughly similar to the already active units.

### M-Wave Duration

The progressive decrease in M-wave duration observed during the low-force contraction could not be due to conduction velocity, as this parameter was seen to remain unchanged. Another possible candidate to explain the narrowing of the M wave, an increase in intramuscular temperature, can also be ruled out: the reason is that Dur_FIRST_ and Dur_SECOND_ decreased markedly during the first 30s of the contraction, while muscle temperature was found remained constant during the same period. We believe that factors related to muscle architecture might have been involved in the reduction of M-wave duration. Specifically, we hypothesize that during the initial phase of the 10% MVC task, the length of muscle fascicles decreased. Our hypothesis is supported by a study of Mademli and Arampatzis (2005), who showed that during an isometric contraction of the plantar flexors at 40% MVC, the fascicle length shortens, this shortening being marked at the initial part of the contraction. Thus, it is conceivable that a muscle fascicle shortening would also occur during a sustained 10% MVC task, since tendon stiffness is lower at low contraction forces (Hodges et al., 2003). In conclusion, the fascicle length would decrease progressively over the first 30s of the contraction as the tendon progressively stretches, leading to a decrease in M-wave duration.

### Amplitude of the M-Wave First Phase

It would be tempting to associate the decrease in Ampli_FIRST_ observed during the contraction to changes in the properties of the sarcolemmal membrane. One such membrane property is conduction velocity: however, impulse conduction remained unchanged during the contraction, and thus could not be involved in the decline of Ampli_FIRST_. Another factor known to have a depressing effect on M-wave amplitude is increased intramuscular temperature (Rutkove, 2000). However, it appears that muscle temperature could not have a major effect on M-wave size: indeed, muscle skin temperature increased marginally during the first 30s of the contraction, whereas most of the decrease in Ampli_FIRST_ occurred precisely during the initial 30s.

A key observation of the study is that Ampli_FIRST_ and Dur_FIRST_ (also Dur_SECOND_ and Dur_PP_) changed in parallel during exercise: namely, all these parameters decreased during the initial 30s of the contraction and then decreased more gradually for the remainder of the contraction. The parallel temporal changes in Ampli_FIRST_ and M-wave duration suggest that these parameters were affected by a common mechanism that occurred during the initial part of the contraction. We believe that a reduction in fascicle length could account for the concurrent decrease in Ampli_FIRST_ and M-wave duration. First, the shortening of muscle fibers is accommodated by bulging of the muscle (Azizi et al., 2008): such bulging likely increased the distance from the fibers to the recording electrode, thus making Ampli_FIRST_ to decrease, as shown by Mesin et al. (2006). In addition, muscle shortening has been shown to produce a decrease in M-wave duration (Rodriguez-Falces and Place, 2014).

### Amplitude of the M-Wave Second Phase

There are two main observations regarding the amplitude of the M-wave second phase. First, we found that Ampli_SECOND_ increased during the contraction, just the opposite to Ampli_FIRST_. The fact that Ampli_FIRST_ and Ampli_SECOND_ changed in opposite directions cannot be solely explained by alterations in fiber membrane properties: indeed, any alteration in the fiber membrane properties would make the first and second M-wave phases change in the same direction (Rodriguez-Falces et al., 2016). Clearly, then, other factors, possibly related to mechanical and/or architectural aspects of the muscle, must be involved.

The second remarkable observation is that, similarly to Ampli_FIRST_, the largest change in Ampli_SECOND_ occurred during the initial 30s of the contraction. The parallel time course of changes in these parameters suggests that they were affected by the same mechanism. Our hypothesis of a shortening of muscle fascicle length could explain the concurrent increase in Ampli_SECOND_ and decrease in Ampli_FIRST_. Certainly, muscle shortening would affect the M-wave second phase, as this phase is generated upon the extinction of the action potentials at the fiber ends (Rodriguez-Falces and Place, 2018). Specifically, a reduction in fascicle length would cause a more synchronous arrival of the action potentials at the tendons, thus provoking an increase in Ampli_SECOND_ (Rodriguez-Falces and Place, 2014). As for the M-wave first phase, the bulging of the muscle caused by muscle shortening would make Ampli_FIRST_ to decrease, as commented above.

### Recovery Period

From our previous studies, we learned that the amplitude of the M-wave first phase is highly sensitive to changes in extracellular K^+^ concentration (for an example, see Rodriguez-Falces and Place, 2017b). After cessation of a prolonged maximal contraction, the rapid and prominent fall in the extracellular K^+^ concentration (for an example see Vyskocil et al., 1983) is accompanied by a fast and marked change in Ampli_FIRST_ (Cupido et al., 1996; Rodriguez-Falces and Place, 2017b). Based on this reasoning, in the present experiments if extracellular K^+^ concentration had dropped markedly after the 10% MVC task, we would have observed a significant change in Ampli_FIRST_ after 5s of rest. However, we found that Ampli_FIRST_ remained unchanged after 5s of rest (also Area_FIRST_ and Dur_FIRST_). This indicates that extracellular K^+^ concentration could not have increased significantly during the 10% MVC task. These results therefore support the observations of Sjøgaard et al. (1988) that, for contraction levels below 10% MVC, blood flow is sufficiently high to maintain K^+^ homeostasis.

Also interesting is the fact that neither of the M-wave parameters returned to control values after the 15-min recovery period (see Figure 5); rather, they remained relatively constant during this recovery time. This in contrast with the progressive recovery of fiber membrane properties observed after prolonged maximal contractions (Van der Hoeven et al., 1993; Cupido et al., 1996). The absence of normalization suggests that the mechanical and/or geometrical changes induced in the muscle and tendon by the low-force contraction might have persisted for a long period after the cessation of exercise, although to date a long-lasting decrease in tendon stiffness has been only demonstrated after repeated high-intensity contractions (Kubo et al., 2001).

### Sarcolemmal Membrane Excitability Was Preserved During the Low-Force Contraction

The present results provide several pieces of evidence suggesting that sarcolemmal excitability was not impaired during the low-force contraction. The most convincing evidence is that conduction velocity, a direct indicator of the membrane polarization state, did not decrease during exercise. Second, Ampli_FIRST_ (the M-wave parameter that most faithfully reflects changes in fiber membrane properties) suffered a moderate decrease (~7%) during the contraction: however, such decline was probably not due to changes in membrane properties, but rather to adjustments in muscle architectural properties (see the above discussion). Third, if membrane excitability was reduced during the contraction, it would be expected that this impairment occurred progressively during the entire length of the task: however, most of the decrease in Ampli_FIRST_ happened within the first 30s. Fourth, if an actual increase in extracellular K^+^ concentration had occurred during the contraction, a rapid fall of this concentration would have happened immediately after exercise: however, we observed no change in Ampli_FIRST_ 5s after the cessation of the contraction. Lastly, based on our previous works, an impairment in sarcolemmal excitability caused by an increase in extracellular K^+^ concentration would be manifested by an increase, and not a decrease, in Ampli_FIRST_ (see Rodriguez-Falces and Place, 2018).

### How Reliable Are the Amplitudes of the First and Second M-Wave Phases to Interpret Changes in Sarcolemmal Excitability?

The present results have shown that a decrease in Ampli_FIRST_ during a low-force contraction can occur even in the absence of changes in fiber membrane excitability. This result is of relevance as it implies that, when a muscle contraction is sustained, there exists “non-sarcolemmal” factors which influence the magnitude of the M-wave first phase. Some of these factors (muscle shortening; electrode-to-fiber distance; intramuscular temperature) have been discussed above. It is important to note that the effects of these “non-sarcolemmal” factors on Ampli_FIRST_ are of limited extent: indeed, the decrease in Ampli_FIRST_ was only of ~7%. It is likely that, when a contraction of moderate or high intensity is sustained, these non-sarcolemmal factors would continue exerting a depressing effect on Ampli_FIRST_, but this effect would be probably counteracted and masked by the increase in extracellular K^+^ concentration, which acts to increase Ampli_FIRST_ (Rodriguez-Falces and Place, 2017b).

The increase in Ampli_SECOND_ observed during first ~30s of the low-force contraction was interpreted to be due to the shortening of muscle fascicles and is consistent with our previous observations (Rodriguez-Falces and Place, 2017a,b). Thus, it appears that even for a very low contraction intensity, muscle shortening exerts a noticeable effect on Ampli_SECOND_. This finding lends support to the idea that the M-wave second phase is highly sensitive to changes in muscle length (Rodriguez-Falces and Place, 2014), and thus this phase should be excluded for the analysis of membrane excitability.

### Limitations of the Study

In the present study we did not measure changes in fascicle length, and thus we can only speculate that the changes observed in M-wave parameters were attributable, to a certain degree, to a shortening in fascicle length. On the other hand, in the present study we adopted a contraction duration of 3min, which was considerably shorter that than the duration (400s) selected by previous investigators (Arendt-Nielsen et al., 1989). This difference in the contraction duration hampers the comparison of the results.

## Conclusion

In conclusion, our results provide solid evidence that sarcolemmal excitability was preserved during a 3-min contraction at 10% MVC force in the *quadriceps*, the most direct evidence being the absence of changes in conduction velocity throughout the contraction.

We found that the amplitude of the first and second phases of the M wave changed in opposite directions during the contraction: the first phase diminished (−7%) for the first ~30s and stabilized thereafter, whereas the second phase enlarged (+7%) for the initial ~30s, before stabilizing. The opposite direction of changes in the first and second phases cannot be solely explained by alterations in fiber membrane properties. This observation, together with the fact that M-wave duration decreased rapidly during the first ~30s, lead us to tentatively suggest that the changes in M-wave parameters were due to the fact that fascicle length decreased progressively over the first 30s as the tendon progressively stretched.

On the other hand, we found that the 3-min contraction at 10% MVC produced muscle fatigue, part of which had a peripheral component, as evidenced by the decrease in the resting twitch, and part of which was due to central mechanisms, as suggested by the disproportionate increase in participants’ perceived effort compared to the increase in EMG_RMS_.

Two additional messages emerged from this study: (1) A depression of the M-wave first phase during a low-force contraction can occur even in the absence of changes in membrane excitability and (2) Development of fatigue during sustained voluntary contractions at low force levels can occur without alteration of membrane excitability.

## Data Availability Statement

The datasets presented in this article are not readily available because the raw data supporting the conclusions of this article will be made available by the authors upon reasonable request. Requests to access the datasets should be directed to javier.rodriguez.falces@gmail.com.

## Ethics Statement

The studies involving human participants were reviewed and approved by Ethics Board of the Public University of Navarra. The patients/participants provided their written informed consent to participate in this study.

## Author Contributions

JR-F and NP designed experimental study. JR-F performed experiments. JR-F analyzed data. JR-F and NP interpreted results of experiments. JR-F drafted manuscript. JR-F and NP edited and revised manuscript. JR-F and NP approved final version of manuscript.

## Funding

This work has been supported by the Spanish Ministry of Science and Innovation under the project PID2019-109062RB-I00.

## Conflict of Interest

The authors declare that the research was conducted in the absence of any commercial or financial relationships that could be construed as a potential conflict of interest.

## Publisher’s Note

All claims expressed in this article are solely those of the authors and do not necessarily represent those of their affiliated organizations, or those of the publisher, the editors and the reviewers. Any product that may be evaluated in this article, or claim that may be made by its manufacturer, is not guaranteed or endorsed by the publisher.

## Supplementary Material

Supplementary Figure S1 Representative traces of EMG signals (a) and force (b) from the *vastus lateralis* (VL), *vastus medialis* (VM), and *rectus femoris* (RF) muscles recorded during the 3min contraction at 10% MVC from one participant.
