# Supplementary figures and images for: Sarcolemmal Excitability, M-Wave Changes, and Conduction Velocity During a Sustained Low-Force Contraction
Source: Front Physiol. 2021 Oct 15;12:732624. doi: 10.3389/fphys.2021.732624 (PMC8554155; doi:10.3389/fphys.2021.732624)

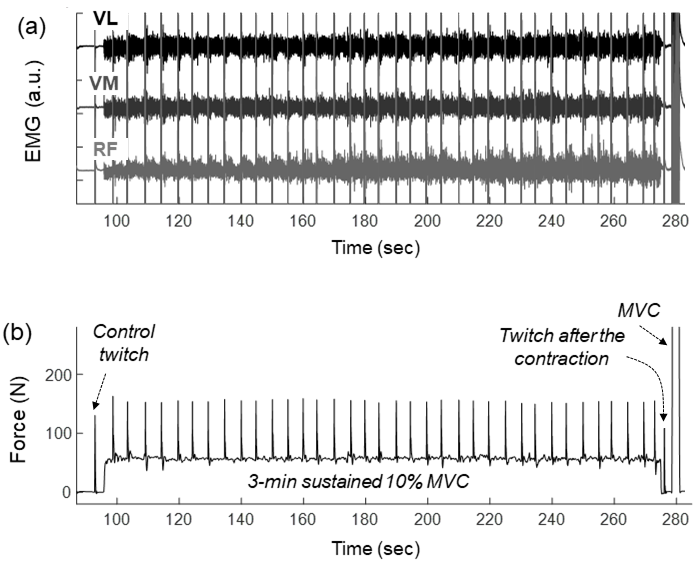

Supplement: Supplementary Figure S1 — Representative traces of EMG signals (a) and force (b) from the vastus lateralis (VL), vastus medialis (VM), and rectus femoris (RF) muscles recorded during the 3min contraction at 10% MVC from one participant. [file Image_1.TIF]
